# Rac1 controls epithelial tube length through the apical secretion and polarity pathways

**DOI:** 10.1242/bio.015727

**Published:** 2015-12-23

**Authors:** Kévin Sollier, Helori-Mael Gaudé, François J.-M. Chartier, Patrick Laprise

**Affiliations:** 1 Département de Biologie Moléculaire, Biochimie Médicale et Pathologie, Université Laval, 9 McMahon, Québec, Québec G1R 3S3, Canada; 2 Centre de recherche sur le cancer, Université Laval, 9 McMahon, Québec, Québec G1R 3S3, Canada; 3 CRCHU de Québec, axe oncologie, 9 McMahon, Québec, Québec G1R 3S3,Canada

**Keywords:** Epithelial tube morphogenesis, Epithelial polarity, *Drosophila* trachea, Rac1, Crumbs, Coracle, Septate junction

## Abstract

The morphometric parameters of epithelial tubes are critical to the physiology and homeostasis of most organs. In addition, many human diseases are associated with tube-size defects. Here, we show that Rac1 limits epithelial tube elongation in the developing fly trachea by promoting Rab5-dependent endocytosis of the apical determinant Crumbs. Rac1 is also involved in a positive feedback loop with the septate junction protein Coracle. Thereby, Rac1 precludes paracellular diffusion and contributes to the septate junction-dependent secretion of the chitin-modifying enzymes Vermiform and Serpentine, which restrict epithelial tube length independently of Crumbs. Thus, Rac1 is a critical component of two important pathways controlling epithelial tube morphogenesis.

## Abstract (precis)

**Summary:** Epithelial tube size regulation sustains organ physiology. Rac1 limits tube elongation in the fly trachea through restriction of apical membrane growth, and by supporting luminal secretion of chitin modifying enzymes.

## INTRODUCTION

Epithelial tubes sustain gas, nutrient and waste exchange to maintain the homeostasis of metazoan tissues. Elucidating the molecular mechanisms specifying tube dimension is essential, as several human pathologies result from tube-size defects. The dorsal trunks of the *Drosophila* tracheal system have emerged as a key *in vivo* model to study size control in multicellular tubular structures (Zuo et al., 2013). Development of dorsal trunks with a precise length and caliber require the assembly of a transient chitin-based luminal extracellular matrix (Tonning et al., 2005; Tsarouhas et al., 2007; Zuo et al., 2013). The secreted chitin-modifying enzymes Vermiform (Verm) and Serpentine (Serp) modulate the mechanical properties of this matrix, thereby preventing tube over-elongation (Devine et al., 2005; Dong et al., 2014; Luschnig et al., 2006; Wang et al., 2006). Mutations affecting many components of the septate junction (SJ, a ladder-like structure precluding transepithelial diffusion) prevent secretion of Verm and Serp, and result in dorsal trunk lengthening (Wang et al., 2006; Wu et al., 2007). Hence, identification of the pathways controlling Verm and Serp trafficking downstream of SJ is an outstanding puzzle to be solved in delineating the molecular mechanisms regulating epithelial tube morphogenesis.

In the fly respiratory system, tube size is defined mainly by the surface area of the apical membrane of tracheal cells (Beitel and Krasnow, 2000; Zuo et al., 2013). The apical transmembrane protein Crumbs (Crb) acts as a crucial apical determinant (Laprise and Tepass, 2011; Tepass et al., 1990; Wodarz et al., 1995). Crb promotes apical membrane growth and elongation of dorsal trunks independently of, and in parallel to, the luminal extracellular matrix pathway (Laprise et al., 2010). Deciphering how Crb activity is controlled in the developing trachea is thus instrumental to further understanding tube-size regulation. The mutually antagonistic relationship between Crb and the small GTPase Rac1 defines apical membrane length in epidermal cells at late stages of *Drosophila* embryogenesis (Chartier et al., 2011, 2012). However, it is unknown whether this functional interplay takes place in tracheal cells, and the role of Rac1 in tubulogenesis remains elusive. Here, we show that Rac1 defines the length of multicellular epithelial tubes by supporting Verm and Serp secretion, and by promoting Crb endocytosis.

## RESULTS AND DISCUSSION

### Rac1 limits Crb activity to define dorsal trunk length

**Fig. 1.**
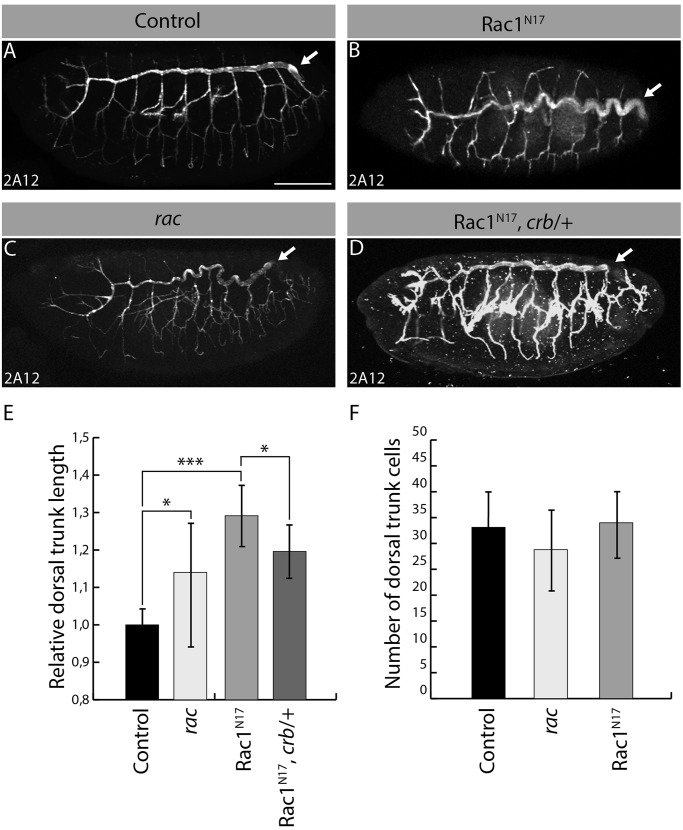
**Impaired Rac1 signaling leads to Crb-dependent over-elongation of dorsal trunks.** (A-D) Immunostaining of the luminal antigen 2A12, which highlights the tracheal tree of embryos (stage 16) of the following genotypes: Control (A: *btl-GAL4,* driver line used to express transgenes in the trachea), Rac1^N17^ (B: *btl-GAL4; UAS-Rac1^N17^*), *rac* (C: *rac1^j11^*, *rac2^Δ^*, *mtl^Δ^*), Rac1^N17^, *crb*/+ (D: *btl-GAL4; UAS-Rac1^N17^*, *crb^11A22^*/+). In each panel, arrow points to a dorsal trunk. Scale bar: 100 µm. (E) Histogram showing the length of dorsal trunks in embryos with altered Rac1 signaling relative to the length of dorsal trunks in control specimens. (F) The number of cells in tracheal segments 7 and 8 was quantified and plotted as a histogram. (E,F) *n*=15 embryos for each genotype, taken from at least three independent experiments. Bars represent mean±s.d. A two-tailed *t*-test was used to evaluate the statistical significance; *P<0.05, ***P<0.001.

To explore the role of Rac1 in tubulogenesis, we expressed a dominant negative form of Rac1 (Rac1^N17^) using the tracheal-specific *btl-GAL4* driver. Embryos expressing Rac1^N17^ established a branched tracheal network similar to control animals (Fig. 1A,B). However, dorsal trunks were over-elongated and convoluted in Rac1^N17^-expressing embryos compared to dorsal trunks seen in control specimens (Fig. 1A,B,E). We observed a similar ectopic lengthening of dorsal trunks in a mutant background with reduced cellular Rac activity (*rac1*, *rac2*, *mtl* zygotic mutants; Ng et al., 2002) (Fig. 1C,E), thus confirming the specificity of the Rac1^N17^-induced phenotype. These data establish that Rac1 is essential to restrict dorsal trunk elongation, thereby contributing to tube-size specification during development. In addition, it was shown previously that a strong expression of Rac1^N17^ (using two copies of the *btl-GAL4* driver) alters cell-cell adhesion and cell intercalation in the developing tracheal tree (Chihara et al., 2003). Thus, Rac1 plays a broad role in epithelial tube morphogenesis. To investigate whether the enlargement of dorsal trunks associated with altered Rac1 signaling results from an increase in cell number or from an enlargement of the surface area of individual cells, we quantified the number of tracheal cells. This analysis reveals that there was no significant variation in dorsal trunk cell numbers in control, Rac1^N17^-expressing or *rac* mutant embryos (Fig. 1F). This implies that reducing Rac1 activity increases the dimension of the apical membrane that faces the lumen and plays a critical role in determining the size of multicellular tubes in the fly trachea (Beitel and Krasnow, 2000; Laprise et al., 2010).

Rac1 is known to limit apical membrane growth by restricting Crb functions in epidermal cells (Chartier et al., 2011). This raises the intriguing possibility that this functional interaction also exists in tracheal cells, and that Crb-dependent apical membrane growth is responsible for tube-size defects upon alteration of Rac1 activity. Accordingly, reduction of *crb* dosage by introducing one copy of a *crb* null allele suppressed dorsal trunk over-elongation in Rac1^N17^-expressing embryos (Fig. 1D,E). Together, these results indicate that Rac1 participates in the apical polarity pathway to limit Crb activity and specify epithelial tube size *in vivo*.

### Rac1 promotes Crb endocytosis, which prevents over-elongation of dorsal trunks

**Fig. 2.**
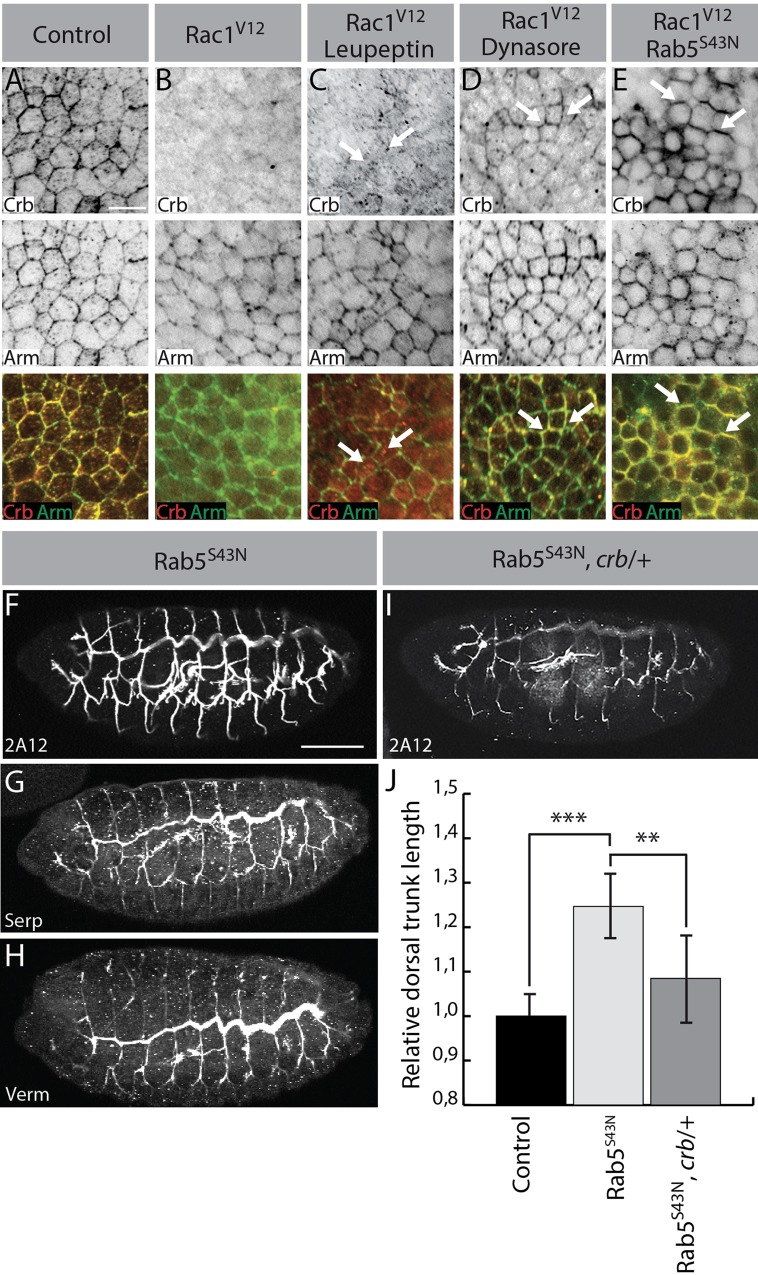
**Rac1 promotes Rab5-dependent endocytosis of Crb.** (A-E) Embryos were co-stained for Crb (red in the merged images) and Arm (green). Panels depict a surface view of the ventral ectoderm of the following embryos: Control (A: *da-GAL4* embryo incubated in DMSO), Rac1^V12^ (B: *da-GAL4/UAS-Rac1^V12^* embryo incubated in DMSO), Rac1^V12^ Leupeptin (C: *da-GAL4/UAS-Rac1^V12^* embryo treated with the lysosomal proteolysis inhibitor leupeptin), Rac1^V12^ Dynasore (D: *da-GAL4/UAS-Rac1^V12^* embryo treated with the dynamin inhibitor dynasore), Rac1^V12^ Rab5^S43N^ (*da-GAL4/UAS-Rac1^V12^*, *UAS-Rab5^S43N^*). Images are representative results of three independent experiments. Scale bar: 10 µm. C, arrows point to intracellular puncta; D,E, arrows indicate cell-cell borders. (F-H) Stage 16 Rab5^S43N^-expressing embryos stained for 2A12 (F), Serp (G) or Verm (H). (I) 2A12 staining performed on an embryo expressing Rab5^S43N^ in the trachea and heterozygous for the null allele *crb^11A22^* (*btl-GAL4; UAS-Rab5^S43N^*/*crb^11A22^*). Scale bar: 100 µm. (J) Histogram showing the quantification of the dorsal trunk length in Rab5^S43N^ (*btl-GAL4; UAS-Rab5^S43N^*) and Rab5^S43N^, *crb*/+ (*btl-GAL4; UAS-Rab5^S43N^*, *crb^11A22^/+*) embryos relative to dorsal trunk length of control animals (*btl-GAL4*). *n*=15 embryos for each genotype, taken from at least three independent experiments. Bars represent mean±s.d. A two-tailed *t*-test was used to evaluate the statistical significance; **P<0.01, ***P<0.001.

Crb protein levels at the apical membrane reflect the balance between Crb delivery, endocytosis and recycling (Blankenship et al., 2007; Lu and Bilder, 2005; Pocha et al., 2011). Expression of a constitutively active form of Rac1 (Rac1^V12^) led to an almost complete loss of Crb, whereas the adherens junction protein Armadillo (Arm) partially remained at the plasma membrane (Fig. 2A,B) (see Chartier et al., 2011; Chihara et al., 2003). It is thus possible that Rac1 modulates Crb trafficking and promotes its degradation. Accordingly, treatment of Rac1^V12^-expressing embryos with the lysosomal proteolysis inhibitor leupeptin resulted in an accumulation of Crb in cytoplasmic puncta in the epidermis (Fig. 2C, arrows; Fig. S1A,B). This shows that Crb is normally degraded in the presence of Rac1^V12^. The increased degradation of Crb caused by ectopic Rac1 activity may reflect an excessive endocytosis of Crb. To investigate this hypothesis, we blocked endocytosis using the dynamin inhibitor dynasore or by expressing a dominant negative form of the early endosome-associated protein Rab5 (Rab5^S43N^) (Entchev et al., 2000). Strikingly, both dynasore and Rab5^S43N^ attenuated the Rac1^V12^-induced loss of Crb (Fig. 2D,E and Fig. S1C). Importantly, Crb remained associated with the plasma membrane in Rac^V12^-expressing embryos with impaired endocytic capacities (Fig. 2D-E, arrows). These results demonstrate that Rac1 controls Crb levels in the epidermis by promoting its endocytosis and degradation. As both the epidermis and the tracheal tree originate from the ectoderm, it is tempting to propose that Rab5 also controls Crb endocytosis in tracheal cells, thereby contributing to tube-size specification.

To investigate the role of Rab5-dependent endocytosis in epithelial tube morphogenesis, we expressed Rab5^S43N^ in tracheal cells. We observed that reduction of Rab5 activity resulted in an over-growth of dorsal trunks without compromising the secretion of Verm and Serp (Fig. 2F-H,J). This confirms previous findings suggesting that Rab5 is required to limit the elongation of multicellular epithelial tubes during development, but is dispensable for formation of SJ (Tsarouhas et al., 2007). Crb is known to be endocytosed through a Rab5-dependent pathway (Harris and Tepass, 2008; Lu and Bilder, 2005). It is thus plausible that Crb accumulation at the apical membrane is responsible for the tube-size defects in embryos with compromised Rab5 activity in the trachea. Indeed, reduction of *crb* dosage in embryos expressing Rab5^S43N^ suppressed over-elongation of dorsal trunks (Fig. 2I,J). Rab5 is thus necessary to prevent the accumulation of Crb at the apical membrane and ectopic growth of dorsal trunks. Rab5 is also required to clear the luminal content at the end of embryogenesis (Tsarouhas et al., 2007), thereby sustaining the liquid-air transition that is essential for the gas exchange function of tracheal tubes. Thus, Rab5-mediated apical endocytosis plays a multifaceted role in the morphogenesis and physiology of the larval respiratory system. Altogether, these data support a model in which Rac1 controls epithelial tube length by promoting Rab5-dependent endocytosis of Crb.

### Rac1 is required for SJ functions as well as for Verm and Serp secretion

**Fig. 3.**
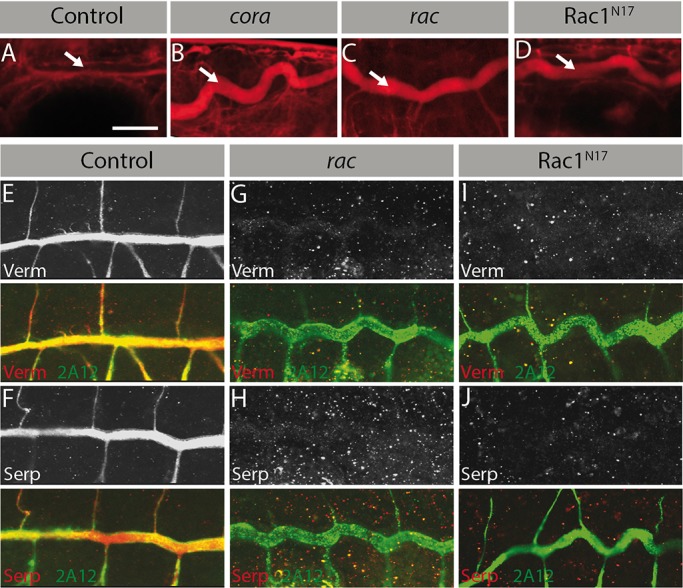
**Rac1 controls SJ permeability and luminal accumulation of chitin-modifying enzymes.** SJ permeability was assessed by injection of fluorescently labeled dextran in control embryos (A: *btl-GAL4*), *cora* mutant embryos (B), *rac* mutant embryos (C: *rac1^j11^*, *rac2^Δ^*, *mtl^Δ^*) or Rac1^N17^-expressing embryos (D: *btl-GAL4; UAS-Rac1^N17^*). In each panel, arrow points to the tracheal lumen. (E-J) Co-staining of Verm and 2A12 (upper panels) or Serp and 2A12 (lower panels) in control (E,F: *btl-GAL4*), *rac* (G,H: *rac1^j11^*, *rac2^Δ^*, *mtl^Δ^*) and Rac1^N17^ (I,J: *btl-GAL4; UAS-Rac1^N17^*) embryos. Scale bar: 30 µm. Six embryos from three independent experiments were analyzed for each genotype.

The SJ-dependent secretion of Verm and Serp plays a critical role in restricting tracheal tube length (Wang et al., 2006). This pathway acts independently of Crb (Laprise et al., 2010). To investigate whether Rac1 also contributes to the SJ-apical secretion pathway, we performed a dye diffusion assay in living embryos. In control animals, the paracellular barrier created by SJ precluded the entry of fluorescent dextran within the dorsal trunk lumen (Fig. 3A, arrow). In contrast, we observed a luminal accumulation of dextran in embryos deficient for the SJ protein Coracle (Cora) (Fig. 3B, arrow), as previously reported (Lamb et al., 1998). Similarly, the dextran clearly leaked within dorsal trunks in *rac* mutant and Rac1^N17^-expressing embryos (Fig. 3C,D), indicating that Rac1 is required for the establishment of a functional SJ. We next examined whether Rac1 is involved in apical secretion of Verm and Serp. While stage 16 control embryos showed strong levels of Verm and Serp in the lumen of tracheal tubes, these enzymes were absent in embryos with altered Rac1 signaling (Fig. 3E-J). However, the level of the luminal antigen 2A12 was normal in *rac* mutant and Rac1^N17^-expressing embryos (Fig. 3G-J). Thus, our data identify Rac1 as a novel regulator of SJ permeability and suggest that Rac1 sustains the apical secretion of selected cargoes, including Verm and Serp.

### Rac1 and Cora are involved in a positive feedback loop

**Fig. 4.**
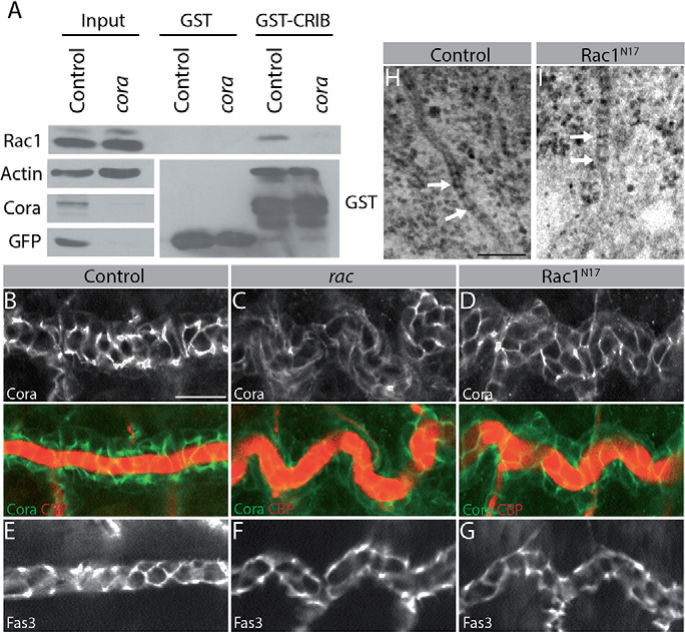
**Rac1 and Cora act in a positive feedback loop.** (A) Control embryos (CyO, *act-GFP*/CyO, *act-GFP* and CyO, *act-GFP*/*cora*) and *cora* mutant embryos (*cora/cora*) were homogenized in Rac1 assay buffer. A fraction of each homogenate was kept to examine Rac1 expression levels (Input), and GST-PAK^CRIB^ (GST-CRIB) was used to pull-down active Rac1. Native GST was employed as negative control. Western blotting was used to detect Rac1, Actin, Cora, GFP and GST. Data show representative images of three independent experiments. (B-D) Immunostaining of Cora and labeling of the apical matrix using the Chitin-Binding Probe (CBP) in control (B: *btl-GAL4*), *rac* (C: *rac1^j11^*, *rac2^Δ^*, *mtl^Δ^*) and Rac1^N17^-expressing (D: *btl-GAL4; UAS-Rac1^N17^*) embryos. Panels show a portion of a dorsal trunk. (E-G) Immunostaining of Fas3 in the dorsal trunk of control (E: *btl-GAL4*), *rac* (F: *rac1^j11^*, *rac2^Δ^*, *mtl^Δ^*) and Rac1^N17^-expressing (G: *btl-GAL4; UAS-Rac1^N17^*) embryos. (B-G) Nine embryos from three independent experiments were analyzed for each genotype. (H,I) Electron microscope micrograph of tracheal cells in control (H: *btl-GAL4*) and Rac1^N17^-expressing (I: *btl-GAL4; UAS-Rac1^N17^*) embryos. *n*=16 for each genotype, taken from a least three independent embryos. Arrows point to septa. Scale bars: 20 µm in A-G; 125 nm in H,I.

So far, our results demonstrate that Rac1 controls both the Crb-polarity and the SJ-apical secretion pathways. Although this is unusual, it is not unique: indeed, Cora controls the permeability of SJ and supports the luminal accumulation of Verm and Serp (Lamb et al., 1998; Laprise et al., 2010). In addition, Cora regulates apical-basal polarity and limits apical membrane growth by restricting Crb functions through unknown mechanisms (Laprise et al., 2009). Reduction of *crb* dosage rescues dorsal trunk defects in *cora* mutant embryos (Laprise et al., 2010), showing that Crb overactivation is the primary cause of dorsal trunk lengthening in the absence of Cora. These observations led us to propose that Rac1 and Cora are functionally linked. Comparison of active Rac1 levels in control and *cora* mutant embryos revealed a decreased amount of GTP-bound Rac1 in Cora-deficient embryos (Fig. 4A), showing that Cora promotes Rac1 activation. Although we haven't directly demonstrated that Cora activates Rac1 in tracheal cells, the similarities between the tracheal phenotypes observed in *cora* mutant and Rac1-deficient embryos argue that Rac1 is a downstream effector of Cora in this tissue. In return, Rac1 could impact on Cora function as our data show that Rac1 is required for formation of functional SJ. To validate this hypothesis, we compared the distribution of Cora in control, *rac* mutant and Rac1^N17^-expressing embryos in dorsal trunk cells. While Cora is concentrated at SJ in control animals, its distribution was more diffuse in embryos with impaired Rac1 signaling (Fig. 4B-D). This misdistribution of Cora is not secondary to altered SJ architecture, as the SJ-associated protein Fas3 maintained a normal localization in embryos mutant for *rac* or expressing the dominant negative form of Rac1 (Fig. 4E-G). In addition, Rac1^N17^-expressing embryos displayed septa that were similar to those formed by control specimens (Fig. 4H,I). Thus, Rac1^N17^ interferes with the localization of Cora, and controls the permeability of SJ without totally disrupting their ultrastructure. This supports the notion that intracellular signaling events can regulate SJ permeability while septa with a normal appearance are present (Laprise et al., 2009). Together, these data show that Rac1 and Cora are linked in a positive feedback loop in which Cora increases the activation level of Rac1, which signals back to Cora to control its appropriate subcellular distribution.

**Fig. 5.**
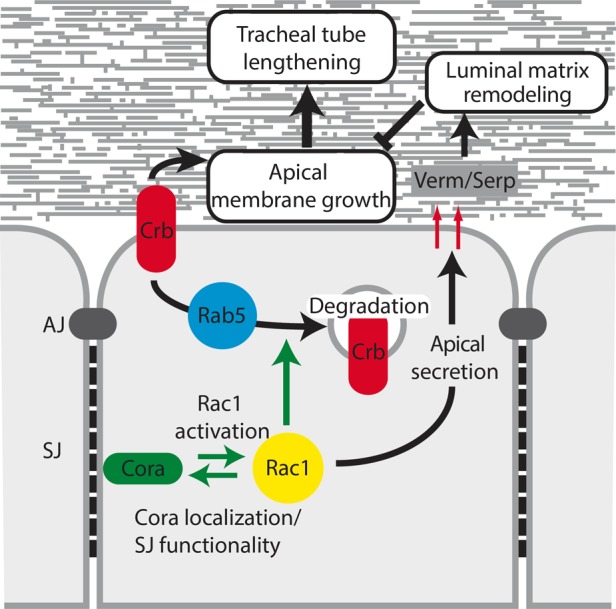
**Model of Rac1 activation and function in the control of tracheal tube length.** Cora activates Rac1-dependent signaling, which supports the luminal accumulation of Verm and Serp. These enzymes are known to modulate the functional properties of the luminal matrix, thereby restricting tube elongation. In return, Rac1 controls Cora localization and SJ permeability. Rac1 also promotes Rab5-dependent endocytosis and subsequent degradation of Crb. This protein favors apical membrane growth and tube lengthening during epithelial tube morphogenesis. Overall, our study establishes that Rac1 acts as a central regulator of tube-size specification.

Overall, our analysis suggests that Rac1 acts as a downstream effector of the SJ-associated protein Cora to promote luminal accumulation of Verm and Serp (Fig. 5). Further exploration of this model will likely shed light on the long-lasting issue of the molecular events linking Cora to apical secretion. Our results also support the intriguing concept that SJ initiates intracellular signaling, as reported for adherens and tight junctions (McEwen et al., 2012; Zihni et al., 2014). In addition, our study positions Rac1 in the molecular network controlling apical secretion, which is essential for tube-size specification and for the physiology of many organs (Zuo et al., 2013). The functional interplay between Cora and Rac1 is bidirectional, as Rac1 promotes the localization of Cora within SJ. Our data also highlight that Rac1 is essential for the SJ-mediated paracellular barrier, likely through its action on Cora. In addition, Rac1 favors Rab5-mediated endocytosis of Crb (Fig. 5), thereby specifying epithelial tube length by preventing over-growth of the apical membrane. This discovery has broad implications. First, it proposes a molecular basis to explain how Cora restricts Crb activity to sustain apical-basal polarity, which is crucial for the morphogenesis and specialized functions of most epithelia (Tepass et al., 2001). Secondly, it provides novel insights into the regulation of Crb levels at the apical membrane, which impact on epithelial tube length (Laprise et al., 2010). The need to further understand tube-size specification is emphasized by the fact that numerous pathologies are associated with tube-size defects (Zuo et al., 2013). Furthermore, the Rac1-dependent regulation of Crb levels may be relevant to cancer, as removal of Crb from the membrane is a critical trigger of epithelial-to-mesenchymal transition (Campbell et al., 2011; Laprise, 2011), which plays a critical role in tumor progression (Thiery et al., 2009). In conclusion, our study demonstrates that Rac1 is a central regulator of epithelial tube morphogenesis.

## MATERIALS AND METHODS

### Drosophila genetics

The alleles used in this work were: *rac1^j11^*, *rac2^Δ^*, *mtl^Δ^* (Ng et al., 2002), *crb^11A22^* (Tepass et al., 1990) and *cora^5^* (Fehon et al., 1994). Recombinant chromosome generated was: *UAS-Rac1^V12^*, *UAS-Rab5^S43N^*. Rac1^N17^ (Luo et al., 1994), Rac1^V12^ (Luo et al., 1994) and Rab5^S43N^ (Entchev et al., 2000) were expressed in fly embryos by crossing the corresponding UAS lines to the ubiquitous driver *da-GAL4* (Wodarz et al., 1995) or the tracheal specific *btl-GAL4* driver (Shiga et al., 1996) at 25°C.

### Antibody production

Polyclonal antibodies against Crb amino acids 867-1048 in fusion with GST were produced in rats.

### Staining of embryos

*Drosophila* embryos were formaldehyde-fixed and processed as described previously (Tepass et al., 1990). For Arm staining, embryos were heat-fixed (Gamblin et al., 2014). Primary antibodies used: mouse anti-2A12 (1:10 dilution; Developmental Studies Hybridoma Bank, DSHB), mouse anti-Cora (1:500; clones C566.9 and C615.16, DHSB), mouse and anti-Tango (1:10; DHSB), mouse anti-Arm (1:250; N2-7A1, DHSB), mouse anti-Fas3 (1:10; 7G10, DHSB), rabbit anti-Verm (1:500; Luschnig et al., 2006), rabbit anti-Serp (1:500; Luschnig et al., 2006), rabbit anti-aPKC (1:250; C-20, Santa Cruz Biotechnology) and rat anti-Crb (1:500; this study). Secondary antibodies were conjugated to Cy3 (Jackson Immunoresearch Laboratories) or Alexa Fluor 488 (Molecular Probes). Rhodamine-conjugated Chitin-Binding Probe (New England BioLabs) was used at a concentration of 4 µg/ml, and co-incubated with secondary antibodies.

### Pharmacological treatment of embryos

Dechorionated embryos were incubated in the dark at 25°C in a solution of 0.9% NaCl (under an octane phase) supplemented with DMSO (control), 0.8 mM Dynasore (30 min; Cedarlane) or 0.1 mg/ml leupeptin (3 h; Bioshop).

### Measurements, quantification of nuclei and statistical analysis

Dorsal trunk (stage 16 embryos) length was measured between tracheal segments 2 to 8 using ImageJ. Average values were normalized to the mean length of dorsal trunks in control embryos [error bars represent standard deviation (s.d.)]. The number of dorsal trunk cells was quantified by counting the number of Tango-positive cells within tracheal segments 7 and 8. A two-tailed *t*-test (*n*=15 embryos) was used to evaluate the statistical significance.

### Septate junction permeability assay

The permeability of dorsal trunks (stage 17 embryos) was assessed as described (Lamb et al., 1998) using a solution of 0.5% Texas Red-conjugated dextran (10 kDa) (Molecular Probes). Specimens were observed by confocal microscopy.

### Analysis of Rac1 activation levels

*cora* mutant embryos were balanced over CyO, *act-GFP*. Stage 14-16 homozygous mutant embryos (GFP negative) were selected using a COPAS Select sorter (Union Biometrica), thus providing an embryo population enriched in *cora* mutants. GFP-positive embryos from the same collection were used as control. Embryos were then homogenized, and active Rac1 was pulled-down and measured as described (Chartier et al., 2012; Picard et al., 2009). 50 µg of embryo lysates were kept to monitor the total amount of Rac1 in each sample by western blotting (Laprise et al., 2002). The following primary antibodies were used: mouse anti-Rac1 (clone 102; BD transduction Laboratories), 1:1000; mouse anti-Cora (clones C566.9 and C615.16; DSHB), 1:500; mouse anti-Actin (Mab1501; EMD Millipore), 1:5000 and rabbit anti-GST (provided by J.-Y. Masson, Laval University), 1:10,000. HRP-conjugated secondary antibodies were used at a 1:1000 dilution (GE Amersham).

### Electron microscopy

Stage 16 embryos were fixed and processed for electron microscopy as described previously (Laprise et al., 2010). Specimens were observed on a JEM-1230 (JEOL) transmission electron microscope.

### Image acquisition and processing

Embryos were imaged in Vectashield (Vector Laboratories) with an Olympus FV1000 confocal system and Fluoview 3.0, using a 40× Apo lens with a numerical aperture of 0.90 or a 20× S Plan Apo lens with a numerical aperture of 0.75. All image acquisition was performed at room temperature, and the brightness/contrast tool in Photoshop was uniformly used to process images.
